# Gould’s Dictionary

**Published:** 1912-04-15

**Authors:** 


### BIBLIOGRAPHY.

GOULD'S DICTIONARY. The sixth revised edition of Gould's Pocket Dictionary, like its predecessors is a wonderfully compact and comprehensive statement of modern medical nomenclature. 34,000 words are briefly defined; and many useful statements in tabular form make this little work a most useful book for students and practitioners. The work is kept up to date by frequent revisions adding greatly to its value. The profession is to be congratulated on this additional facility and for so much accurate information made so conveniently accessible.

